# A comparison of five malaria transmission models: benchmark tests and implications for disease control

**DOI:** 10.1186/1475-2875-13-268

**Published:** 2014-07-10

**Authors:** Dorothy I Wallace, Ben S Southworth, Xun Shi, Jonathan W Chipman, Andrew K Githeko

**Affiliations:** 1 Department of Mathematics, Dartmouth College, Hanover, NH, USA; 2 Department of Applied Mathematics, University of Colorado, Boulder, CO, USA; 3 Department of Geography, Dartmouth College, Hanover, NH, USA; 4 Kenya Medical Research Institute, Kisumu, Kenya

**Keywords:** Malaria, Epidemiology, Mathematical model, Sensitivity analysis, Disease control, Entomological inoculation rate

## Abstract

**Background:**

Models for malaria transmission are usually compared based on the quantities tracked, the form taken by each term in the equations, and the qualitative properties of the systems at equilibrium. Here five models are compared in detail in order to develop a set of performance measures that further illuminate the differences among models.

**Methods:**

Five models of malaria transmission are compared. Parameters are adjusted to correspond to similar biological quantities across models. Nine choices of parameter sets/initial conditions are tested for all five models. The relationship between malaria incidence in humans and (1) malaria incidence in vectors, (2) man-biting rate, and (3) entomological inoculation rate (EIR) at equilibrium is tested for all models. A sensitivity analysis for all models is conducted at all parameter sets. Overall sensitivities are ranked for each of the five models. A set of simple control interventions is tested on two of the models.

**Results:**

Four of these models behave consistently over a set of nine choices of parameters and initial conditions, with one behaving significantly differently. Two of the models do not match reported entomological inoculation rate data well. The sensitivity profiles, although consistently having similar top parameters, vary not only between models but among choices of parameters and initial conditions. A numerical experiment on two of the models illustrates the effect of these differences on control strategies, showing significant differences between models in predicting which of the control measures are more effective.

**Conclusions:**

A set of benchmark tests based on performance measures are developed to be used on any proposed malaria transmission model to test its overall behaviour in comparison to both other models and data sets.

## Background

Efforts to control all strains of malaria always include steps to reduce contact between the mosquito vector and humans, including the introduction of bed nets, adulticide, and larvicide. Mathematical models are the key to understanding optimal delivery of these interventions, especially regarding timing of adulticide and larvicide applications, often yielding non-intuitive results for vector borne disease, as in Baumrin *et al.*[1]. Numerical experiments can also test the effects of multiple simultaneous interventions, the closest one can come to a controlled experiment.

A recent paper by Mandal *et al.*[2] describes an assortment of models of malaria, descended from an early nonlinear system of ordinary differential equations of Ross [3]. Smith *et al.*[4] and Reiner *et al.*[5] trace the development of descendants of the Ross model and the increasing complexity in these models over the last century. Dynamic models given by systems of ordinary differential equations have an advantage over models based on statistical correlations or data fitting. Dynamic models take as input the parameters controlling the spread of disease, producing predicted incidence of malaria cases as output, evolving over time. These dynamic systems attempt to model the mechanisms driving malaria dynamics. Consequently, variations in input such as bite rate, initial incidence of disease, prevalence of mosquitoes can, therefore, be explored to see their effects on malaria prevalence in the model. Causality is clearer in these dynamic models than in data-driven correlations.

Unfortunately, dynamical models for malaria transmission are rarely validated against real world data sets. Gleaning parameters from a variety of sources is the usual approach for modelling because mathematicians do not have resources to collect the data they actually need to support their model. Thus Chitnis *et al.*[6] use estimated equilibrium mosquito population from Western Kenya, bite rates from Khmer and New Guinea, mosquito-to-human transmission probabilities from a paper that estimates these from an earlier publication in 1974, and human-to-mosquito transmission probabilities from Africa. The parameters do not represent data collected in the same year, at the same location, or even for necessarily the same species of mosquito. Thus the results of this paper, as of those that came before, are largely qualitative. Judged only by their qualitative behaviour, the five models chosen for in-depth comparison show some useful similarities as well as annoying discrepancies that must be resolved if one is to hope for quantitatively accurate results.

However, it is still possible to compare different models based on overall dynamics and response to parameter changes. To the extent that multiple models tell the same story, it is possible to draw limited conclusions about the effectiveness of control measures under different scenarios. As a combination of control measures (bed nets, spraying, larvicide, patient treatment) are likely to be necessary to manage or eradicate malaria in regions where it is endemic, the relative sensitivity of a model to corresponding parameter changes (bite rate, mosquito death rate, mosquito production rate, human recovery rate, respectively) is a critical piece of the puzzle. In this paper the performance, and especially the sensitivities, of five models are compared in order to see what implications these models, taken together, may have for malaria control.

Surveys of models usually include comparisons of structure and bifurcation properties. While useful, these comparisons do not necessarily capture comparisons of greatest concern in practice, such as the relationships between 1) disease incidence in vectors and humans, 2) human disease *versus* man-biting rate and entomological inoculation rate, and 3) response to treatment interventions. The tests developed here are constructed, not from the point of view of a developer of models, but from the perspective of potential implementation. The numerical experiments described in this paper allow models to be compared on the basis of their performance on measures of specific relevance to malaria control, complementing comparisons based on their structure.

## Methods

### Overview of models chosen

Five models were chosen for comparison. The original Ross [3] and Macdonald [7] models were selected for their simplicity, Chitnis [6] and McKenzie [8] as more complex models, and Anderson/May [9] as an intermediate example. All of the models include populations of susceptible and infected humans and mosquitoes. The Ross and model includes only those populations, while the Macdonald model adds exposed mosquitoes. The other three add exposed humans and mosquitoes as well. McKenzie and Chitnis include the recovered human population, but their definitions of “recovered” differ. In Chitnis, the “recovered” population includes individuals who are temporarily immune and still infectious. In McKenzie, the “recovered” population includes individuals who are temporarily immune but not infectious. Anderson/May includes time delays for progression from exposed to infected for both humans and mosquitoes. One version of McKenzie’s model also incorporates a time delay, but the one tested here does not.

For certain parameters each of these models has a stable disease free equilibrium. Similarly, for some parameters the disease free equilibrium becomes unstable and a stable endemic equilibrium appears. The greater complexity of the Chitnis and McKenzie models is due to the form of various terms in the equations, rather than the number of compartments in the model. The Chitnis model has certain parameters for which the endemic equilibrium enters at a high value, and is more complex than the others, not only in its structure but in its bifurcation behaviour.

How overall population size was set and controlled is one source of variation among models. Chitnis is the only model that incorporates dynamic human and mosquito population size, where *N*_*h*_ is the total human population and *N*_*m*_ the total mosquito population. All other models assume a constant population for humans and mosquitoes. In the McKenzie model, mosquito populations are fractional with respect to the total human population’s size, i.e. if *I*_*m*_=2 in McKenzie, this means that there are twice as many infected mosquitoes as the entire human population. In the other four models all human variables are fractional, where *s*_*h*_ is the proportion of humans who are susceptible, and so on. McKenzie’s model is stated in terms of proportional human population values *S*_*h*_, *I*_*h*_, *etc.*, but the mosquito populations were converted to proportions of total mosquito population for the purpose of comparison with the other four models.

**Figure 1:**
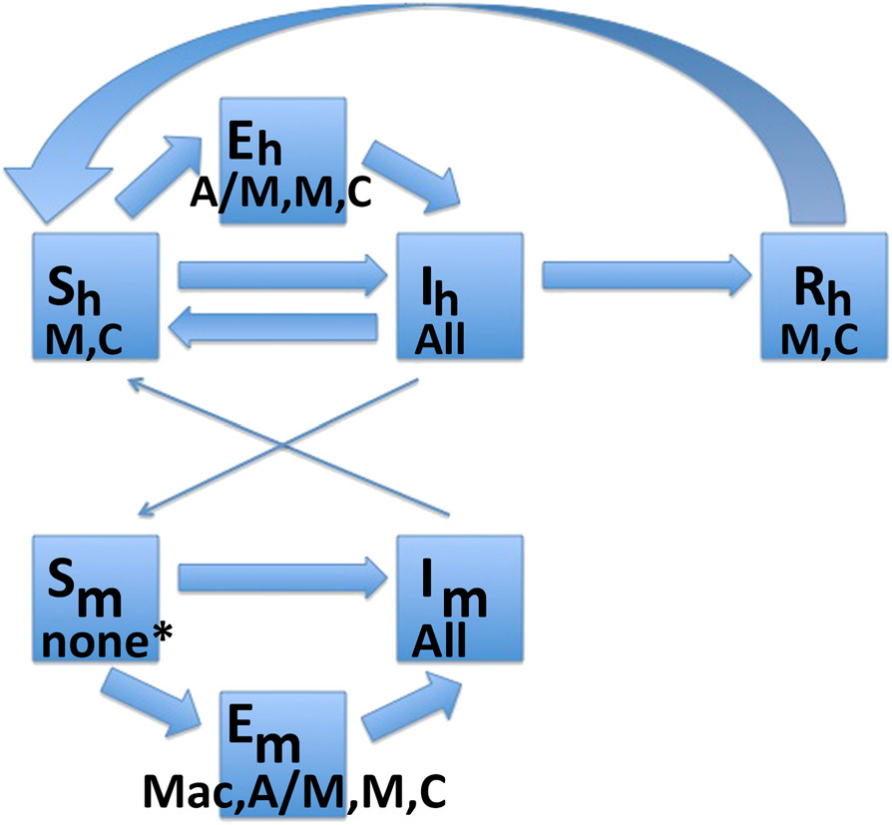
**Schematic describing all five models.** Quantities represented in the Ross, Macdonald, Anderson/May, McKenzie and Chitnis models of malaria transmission. Arrows indicate directions of flow when a quantity is present in the model. R = Ross, Mac = Macdonald, A/M = Anderson/May, M = McKenzie, C = Chitnis. *Susceptible mosquitoes are not included directly in any model, but Chitnis includes the total mosquito population, so susceptible mosquitoes are implicitly modeled.

State variables of all models were reinterpreted as proportions when necessary for purposes of comparing models. Variables *e*_*h*_, *i*_*h*_ and *r*_*h*_ represent the proportion of the human population which is exposed, infected and recovered, respectively. Similarly, *e*_*m*_ and *i*_*m*_ represent the proportion of the mosquito population which is exposed and infected. Figure 1 summarizes the models visually. A mathematical summary of the five models is given in Table 1.

**Table 1:** Expressions for derivatives in all models

	**Ross**	**Macdonald**	**McKenzie**
*S*_*h*_	-	-	*qR*_*h*_(*t*)-*hI*_*m*_(*t*)*S*_*h*_(*t*)
*e*_*h*_,*E*_*h*_	-	-	*hI*_*m*_(*t*)*S*_*h*_(*t*)-*kE*_*h*_(*t*)
*i*_*h*_,*I*_*h*_	*abmi*_*m*_(*t*)(1-*i*_*h*_(*t*))	*abmi*_*m*_(*t*)(1-*i*_*h*_(*t*))	*kE*_*h*_(*t*)-*pI*_*h*_(*t*)
-	-*ri*_*h*_(*t*)	-*ri*_*h*_(*t*)	
*r*_*h*_,*R*_*h*_	-	-	*pI*_*h*_(*t*)-*qR*_*h*_(*t*)
*S*_*m*_	-	-	*f*-*hI*_*h*_(*t*)*S*_*m*_(*t*)-*dS*_*m*_(*t*)
*e*_*m*_,*E*_*m*_	-	*aci*_*h*_(*t*)(1-*e*_*m*_(*t*)-*i*_*m*_(*t*))-*μ*_2_*e*_*m*_(*t*)	*hI*_*h*_(*t*)*S*_*m*_(*t*)-*gE*_*m*_(*t*)-*dE*_*m*_(*t*)
-	-	$-{\text{aci}}_{h}(t-\tau_{m})\left(\right)1-e_{m}(t-\tau_{m})-i_{m}(t-\tau_{m})))e^{-\mu_{2}\tau_{m}}$	
*i*_*m*_,*I*_*m*_	*aci*_*h*_(*t*)(1-*i*_*m*_(*t*))	*aci*_*h*_(*t*-*τ*_*m*_)(1-*e*_*m*_(*t*-*τ*_*m*_)-*μ*_2_*i*_*m*_(*t*)	*gE*_*m*_(*t*)-*dI*_*m*_(*t*)
-		$-i_{m}(t-\tau_{m})))e^{-\mu_{2}\tau_{m}}$	
		**Anderson/May**	
*e*_*h*_,*E*_*h*_		$-{\text{abmi}}_{m}(t-\tau_{h})\left(\right)1-e_{h}(t-\tau_{h})-i_{h}(t-\tau_{h})))e^{-\tau_{h}(r+\mu_{1})})$	
*i*_*h*_,*I*_*h*_		*abmi*_*m*_(*t*-*τ*_*h*_)(1-*e*_*h*_(*t*-*τ*_*h*_)	
		$-i_{h}(t-\tau_{h})))e^{-\tau_{h}(r+\mu_{1})}-{\text{ri}}_{h}(t)-\mu_{1}i_{h}(t)$	
*e*_*m*_,*E*_*m*_		*aci*_*h*_(*t*)(1-*e*_*m*_(*t*)-*i*_*m*_(*t*))-*μ*_2_*e*_*m*_(*t*)	
-		$-{\text{aci}}_{h}(t-\tau_{m})\left(\right)1-e_{m}(t-\tau_{m})-i_{m}(t-\tau_{m})))e^{-\mu_{2}\tau_{m}}$	
*i*_*m*_,*I*_*m*_		*aci*_*h*_(*t*-*τ*_*m*_)(1-*e*_*m*_(*t*-*τ*_*m*_)-*μ*_2_*i*_*m*_(*t*)	
-		$-i_{m}(t-\tau_{m})))e^{-\mu_{2}\tau_{m}}$	
		**Chitnis**	
*S*_*h*_		*qR*_*h*_(*t*)	
		-*hI*_*m*_(*t*)*S*_*h*_(*t*)	
*e*_*h*_,*E*_*h*_		$\left(\right)\frac{\sigma_{m}\sigma_{h}N_{m}(t)b_{\text{hm}}i_{m}(t)}{\sigma_{m}N_{m}(t)+\sigma_{h}N_{h}(t)}))\left(\right)1-e_{h}(t)-i_{h}(t)-r_{h}(t)))$	
		$-\left(\right)\nu_{h}+\psi_{h}+\frac{\Lambda_{h}}{N_{h}(t)}))e_{h}(t)+\delta_{h}i_{h}(t)e_{h}(t)$	
*i*_*h*_,*I*_*h*_		$\nu_{h}e_{h}(t)-\left(\right)\gamma_{h}+\delta_{h}+\psi_{h}+\frac{\Lambda_{h}}{N_{h}(t)}))i_{h}(t)$	
		+*δ*_*h*_*i*_*h*_(*t*)^2^	
*r*_*h*_,*R*_*h*_		$\gamma_{h}i_{h}(t)-\left(\right)\rho_{h}+\psi_{h}+\frac{\Lambda_{h}}{N_{h}(t)}))r_{h}(t)+\delta_{h}i_{h}(t)r_{h}(t)$	
*N*_*h*_		*Λ*_*h*_+*ψ*_*h*_*N*_*h*_(*t*)-(*μ*_1*h*_+*μ*_2*h*_*N*_*h*_(*t*))*N*_*h*_(*t*)-*δ*_*h*_*i*_*h*_(*t*)*N*_*h*_(*t*)	
*e*_*m*_,*E*_*m*_		$\left(\right)\frac{\sigma_{m}\sigma_{h}N_{h}(t)}{\sigma_{m}N_{m}(t)+\sigma_{h}N_{h}(t)}))\left(\right)b_{\text{mh}}i_{h}(t)+{\tilde{b}}_{\text{mh}}r_{h}(t)))\left(\right)1-e_{m}(t)-i_{m}(t)))$	
*i*_*m*_,*I*_*m*_		*ν*_*m*_*e*_*m*_(*t*)-*ψ*_*m*_*i*_*m*_(*t*)	
*N*_*m*_		*ψ*_*m*_*N*_*m*_(*t*)-(*μ*_1*m*_+*μ*_2*m*_*N*_*m*_(*t*))*N*_*m*_(*t*)	

### Parameters and initial conditions

Models were tested for nine sets of parameters and initial conditions. Three sets of initial conditions express a variety of possible situations: midrange human prevalence and high vector prevalence, higher human prevalence and low vector prevalence, and low prevalence in both populations. Note that in some of the models the authors compute some parameter values based on initial conditions, and their method was followed here. The parameter *m*, the mosquito to human ratio in the Ross, Macdonald, and Anderson/May models, is computed from initial conditions. For all models, the man-biting rates and entomological inoculation rate are dependent on mosquito/human ratios set by initial conditions.

Neither the Ross or Macdonald model include all of these groups as variables. For those in which recovered is not a human category, the initial recovered population was included in the susceptible group. If there is not an exposed category in humans and/or mosquitoes, the initial exposed population was combined with the infected group because the mosquitoes or humans have already contracted the disease.

Parameter values across models, listed in Tables 2, and 3, were made as consistent as possible using the relationships described in the caption of Table 2. Chitnis *et al.*[6] was the primary source for parameter values, as they provided the greatest depth in citation and explanation of values used. Parameter set 1 represents the highest transmission intensity (high bite rate, low mosquito mortality), while parameter set 2 represents the lowest. Parameter set 3 has a high bite rate but also high mosquito mortality. Taken together with the various initial conditions the nine scenarios provide a variety of transmission rates and mosquito to human ratios. There is enough variety in the initial conditions to approximate the data in Beier *et al.*[10], at least for some of the models.

**Table 2:** Summary of all parameters included

**Label, Units**	**Description**	**Models used in**
*Λ*_*h*_, Humans/Day	Immigration rate of humans	C
*ψ*_*h*_, Day ^-1^	Per capita birth rate of humans	C
* *ψ*_*m*_, Day ^-1^	Per capita birth rate of mosquitoes	C
*f, Day ^-1^	Mosquito natality	M
*a, Day ^-1^	Man biting rate	AM, Mac R
*h, Day ^-1^	Daily rate of mosquito biting	M
* *σ*_*m*_, Day ^-1^	Number of times a mosquito would bite a human if humans were freely available	C
*σ*_*h*_, Day ^-1^	Maximum number of mosquito bites a human can have	C
*b*, -	Proportion of bites which produce infection in humans	AM, Mac, R
*b*_*h* *m*_, -	Probability of transmission of infection from an infectious mosquito to a susceptible human	C
*c*, -	Proportion of bites by which a susceptible mosquito is infected	AM, Mac, R
*b*_*m* *h*_, -	Probability of transmission of infection from an infectious human to a susceptible mosquito	C
${\tilde{b}}_{\text{mh}}$, -	Probability of transmission of infection from a recovered (asymptomatic carrier) human to a susceptible mosquito	C
*m, -	Ratio of number of female mosquitos to that of humans	AM, Mac, R
*ν*_*h*_, Day ^-1^	Per capita rate of human progression from exposed to infectious	C
*# *ν*_*m*_, Day ^-1^	Per capita rate of mosquito progression from exposed to infectious	C
*# *τ*_*h*_, Day	Latent period of human	AM
*τ*_*m*_, Day	Latent period of mosquito	AM, Mac
*DM*, Day	Length of the interval between mosquito infection and the onset of infectivity	M
*DH*, Day	Length of the interval between human infection and the onset of infectivity	M
*WN*, Day	Duration of a host’s infectivity to vectors	M
*r*, Day ^-1^	Average recovery rate of humans	AM, Mac, R
*γ*_*h*_, Day ^-1^	Per capita recovery rate of humans	C
*ρ*_*h*_, Day ^-1^	Per capita rate of loss of immunity for humans	C
*IM*, Day ^-1^	A human’s susceptibility to re-infection through daily decay of immunity	M
*δ*_*h*_, Day ^-1^	Per capita disease induced death rate for humans	C
*d*, Day ^-1^	Mosquito mortality	M
*μ*_1_, Year ^-1^	Per capita rate of human mortality AM	
* *μ*_2_, Day ^-1^	Per capita rate of mosquito mortality	AM, Mac, R
*μ*_1*h*_, Day ^-1^	Density independent part of death (and emigration) rate of humans	C
*μ*_2*h*_, Humans ^-1^ x Day ^-1^	Density dependent part of death (and emigration) rate of humans	C
*μ*_1*m*_, Year ^-1^	Density independent part of death rate of mosquitoes	C
* *μ*_2*m*_, Mosquito ^-1^ x Day ^-1^	Density dependent part of death rate of mosquitoes	C
*k*,*p*,*q*, Day ^-1^	Human flow rates from Exposed to Infected, Infected to Recovered, and Recovered to Susceptible, respectively	M
*#*g*, Day ^-1^	Mosquito flow rate from Exposed to Infected	M

KEY: *depends on mosquito population and dynamics, #depends on temperature.

**Table 3:** Sets of parameter values used in numerical experiments

**Model**	**Parameters**	**Parameter set 1**	**Parameter set 2**	**Parameter set 3**
**McKenzie**	**DH, DV, WN, IM,**	**0, 15, 30,.009,**	**25, 10, 25,.0027,**	**15, 5, 15,.00055,**
	**h, d, f**	**.3,.03302,.13**	**.1,.03304,.05**	**.5,.1,.15**
Ross	a, b, c, r, *μ*_2_	.3,.2,.5,.01,.03302	.1,.03,.275,.0035,.03304	.5,.4,.4,.05,.1
Macdonald	a, b, c,	.3,.2,.5,	.1,.03,.275,	.5,.4,.4,
	r, *μ*_2_, *τ*_*m*_	.01,.03302, 15	.0035,.03304, 10	.05,.1, 5
Anderson	a, b, c, r,	.3,.2,.5,.01,	.1,.03,.275,.0035,	.5,.4,.4,.05,
and May	*μ*_1_, *μ*_2_, *τ*_*h*_, *τ*_*m*_	.017/365,.033, 10, 15	5.2·10^-6^,.033, 25, 10	1.03·10^-5^,.1, 15, 5
Chitnis	*Λ*_*h*_, *ψ*_*h*_, *ψ*_*m*_, *σ*_*m*_,	.033, 1.1·10^-5^,.13,.5,	.041, 5.5·10^-5^,.05,.3, 5,.	.037,.00011,.15,.6,
	*σ*_*h*_, *b*_*h* *m*_, *b*_*m* *h*_, ${\tilde{b}}_{\text{mh}}$,	19,.2,.5,.048,	03,.25,.025,.04,	3.5,.4,.37,.03,
	*ν*_*h*_, *ν*_*m*_,	.1,.067,	.1,.0035,	.067,.2,
	*γ*_*h*_, *δ*_*h*_,	.01, 9·10^-5^,	2·10^-5^,.0027,	.05, 7·10^-5^,
	*ρ*_*h*_, *μ*_1*h*_, *μ*_2*h*_,	.009, 4.6·10^-5^, 5·10^-7^,	5·10^-6^, 2·10^-7^,	.00055, 10^-5^, 3·10^-7^,
	*u*_1*v*_, *u*_2*v*_	033, 2·10^-5^	.033, 4·10^-5^	.1-2·10^-5^, 2·10^-5^
Initial	Quantity	High	Medium	Low
condition		transmission	transmission	transmission
	*S*_*h*_,*E*_*h*_,*I*_*h*_	500,10,30	510,50,40	600,20,3
	*S*_*m*_,*E*_*m*_,*I*_*m*_	4000,100,50	3000,10,25	2400,30,5

Relationships among parameters: *b*=*b*_*h* *m*_, $c=b_{\text{mh}}+{\tilde{b}}_{\text{mh}}$, $\tau_{h}=\text{DH}=\frac{1}{\nu_{h}}$, $\tau_{m}=\text{DM}=\frac{1}{\nu_{m}}$, *r*=*γ*_*h*_, *μ*_1_=*μ*_1*h*_+*μ*_2*h*_, *μ*_2_=*μ*_1*m*_+*μ*_2*m*_, *h*=*a*, *d*=*μ*_1_=1-*VS*, *q*=*IM*=*ρ*_*h*_, $p=\frac{\text{log}2}{\text{WN}}$, $k=\frac{\text{log}2}{\text{DH}}$, $g=\frac{\text{log}2}{\text{DV}}$, *ψ*_*m*_=*f*. Notes on initial conditions: For those in which recovered is not a human category, the initial recovered population was included in the susceptible group. If there is not an exposed category in humans and/or mosquitos, the initial exposed population was combined with the infected group. Absolute numbers were reinterpreted as percentages or ratios when appropriate.

### Checks for consistency

**Figure 2:**
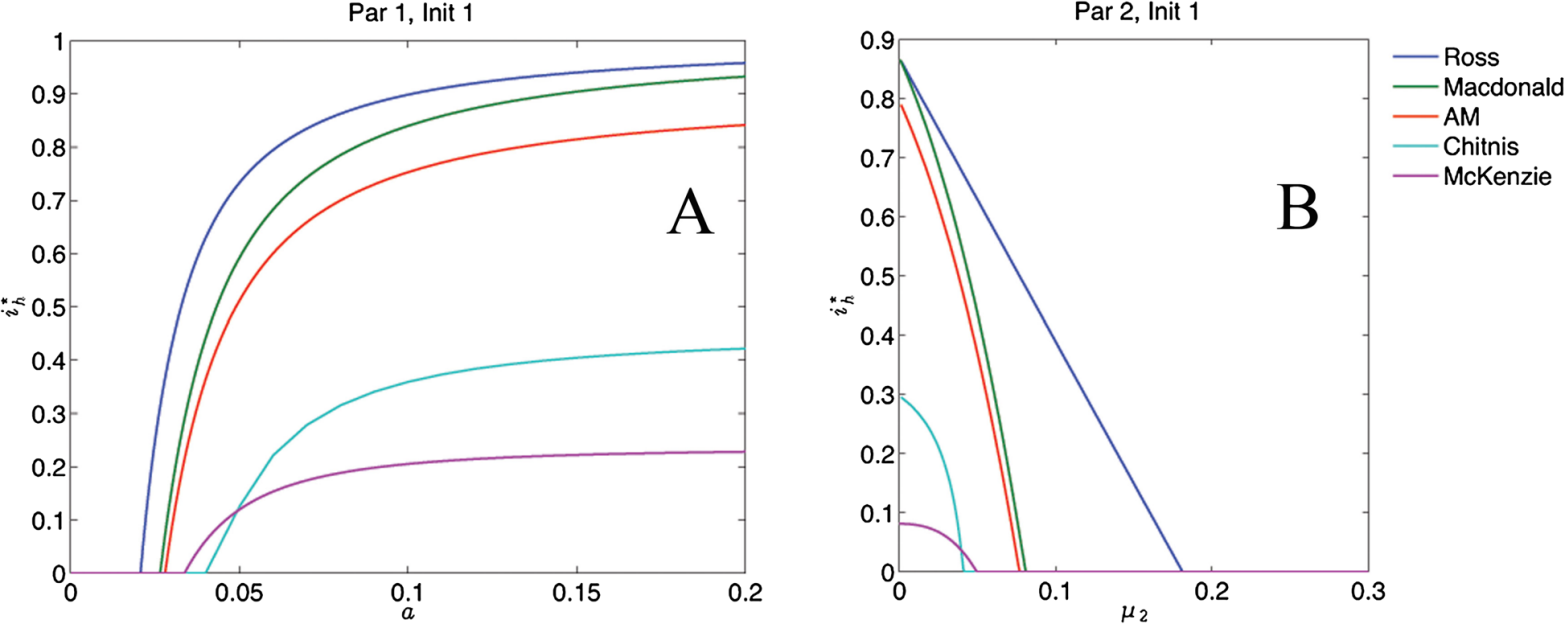
**Bifurcation diagram of**$i_{h}^{*}$***versus* A) biting rate*a* for parameter set 1 and the first set of initial conditions; and B) mosquito mortality rate*μ***_**2**_** for parameter set 2 and the first set of initial conditions, for all five models.** Because all five models are pictured at once, the unstable branch of the bifurcation given by the disease-free equilibrium is not pictured. Note that the ordering of magnitude is preserved for the range in which the parameter is varied, except for the McKenzie and Chitnis models.

**Figure 3:**
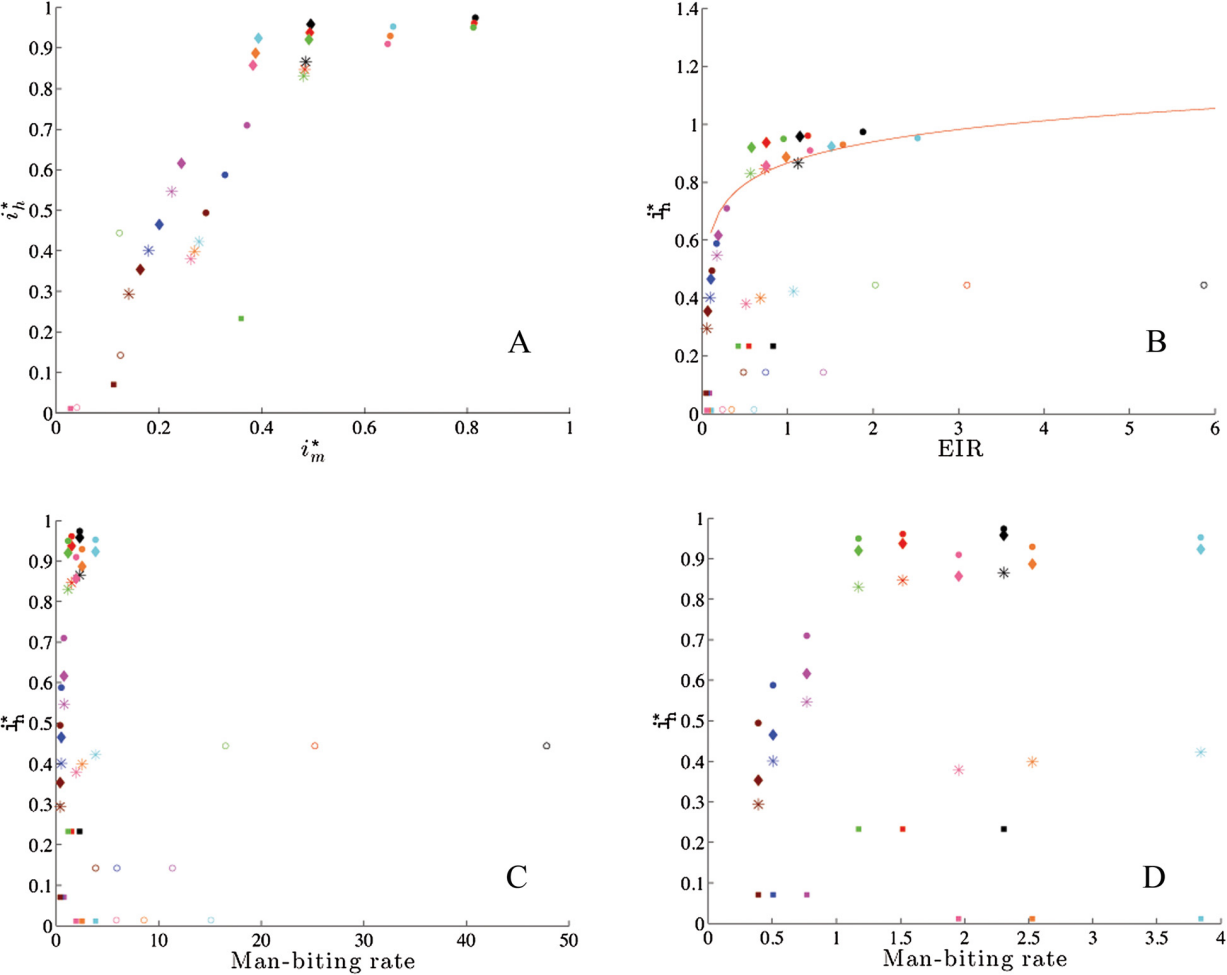
**A series of model comparisons.** These panels describe summary data for equilibrium performance of all five models across all nine sets of parameters and initial conditions. The data displays both general trends and outliers to these trends. **A.** Infected human *versus* infected mosquito equilibrium proportions are plotted for models. **B.** Equilibrium values of infected humans *versus* entomological inoculate rate (EIR): Malaria prevalence at equilibrium is plotted against the equilibrium value of the entomological inoculate rate. The curve is given by Beier *et al.*[10] based on a review of field studies. **C.** and **D.** Equilibrium values of infected humans *versus* man-biting rate: On the left $i_{h}^{*}$ is plotted *versus* the man-biting rate for all models. On the right the Chitnis model is removed. Key: Ross(dots), Macdonald (diamonds), Anderson and May (astirisks), and McKenzie (squares), Chitnis (open circles).

Reproduction numbers were calculated for each of the nine variations and were found to be consistent with assumed transmission intensity across all models. Time series outputs for all 45 systems were checked for consistency. The parameter sets and initial conditions intended to describe lower transmission systems did produce lower disease prevalence, across all models. All simulations were done with Matlab software.Bifurcation diagrams for bite rate and mosquito mortality were created for all 45 simulations to check that all systems exhibited the correct behaviour around the critical value where the disease free equilibrium becomes unstable. Two examples of bifurcation diagrams for bite rate and mosquito mortality are shown in Figure 2.For each scenario and all five models, the proportion of infected humans was plotted against the proportion of infected mosquitoes at equilibrium, shown in Figure 3A. Human disease prevalence is plotted against entomological inoculation rate in Figure 3B, and against man-biting rate in Figures 3C and 3D. These plots check for the agreement of the models with intuition (in the case of 3A, 3C and 3D) and data (3B).

### Sensitivity analysis

Sensitivities, to all parameters of infected vector and human populations at equilibrium, were tested for all models and parameter sets. Equilibrium was established with the original parameter values, and then recalculated for each parameter adjustment. For each pair of parameters and initial conditions, the changes in $i_{m}^{*}$ and $i_{h}^{*}$ caused by an increase or decrease in any given parameter was calculated, and parameters were ranked in order of overall sensitivity in human and mosquito populations using two metrics. For simpler models (Ross, MacDonald, McKenzie) this calculation was done analytically and checked for agreement with computational results. For the more complex models (Anderson/May and Chitnis) numerical experiments were done using Matlab software.

Starting from values given in Tables 4 and 5 (nine total runs for each model), each parameter was adjusted plus or minus 5% while keeping all others constant. A 5% change in a given parameter may correspond to an error in measurement or may represent an intervention that affects the parameter. Although a crude description of sensitivity, it is a realistic description of a measurable change in the parameter itself.

**Table 4:** Sensitivity rankings: two rankings given, top five parameters in order

Ross	Method 1 infected	(*a*,*r*,*b*,*μ*_2_,*c*)
	humans only	
	Method 1 infected	(*a*,*μ*_2_,*c*,*r*,*b*)
	mosquitoes only	
	Method 2 infected	(*a*,*r*,*b*,*μ*_2_,*c*)
	humans only	
	Method 2 infected	(*a*,*μ*_2_,*c*,*r*,*b*)
	mosquitoes only	
MacDonald	Method 1 infected	(*a*,*r*,*μ*_2_,*b*,*τ*_*m*_)
	humans only	
	Method 1 infected	(*μ*_2_,*a*,*τ*_*m*_,*c*,*r*)
	mosquitoes only	
	Method 2 infected	(*a*,*r*,*b*,*μ*_2_,*τ*_*m*_)
	humans only	
	Method 2 infected	(*μ*_2_,*a*,*τ*_*m*_,*c*,*r*)
	mosquitoes only	
Anderson/May	Method 1 infected	(*r*,*a*,*τ*_*h*_,*μ*_2_,*b*,)
	humans only	
	Method 1 infected	(*μ*_2_,*a*,*c*,*τ*_*m*_,*r*)
	mosquitoes only	
	Method 2 infected	(*r*,*a*,*τ*_*h*_,*μ*_2_,*b*)
	humans only	
	Method 2 infected	(*μ*_2_,*a*,*c*,*τ*_*m*_,*r*)
	mosquitoes only	
McKenzie	Method 1 infected	(*h*,*IM*,*DH*,*WN*,*f*_1_)
	humans only	
	Method 1 infected	(*DH*,*f*_1_,*d*,*WN*,*IM*)
	mosquitoes only	
	Method 2 infected	(*h*,*IM*,*WN*,*DH*,*f*_1_)
	humans only	
	Method 2 infected	(*f*_1_,*DH*,*WN*,*d*,*IM*)
	mosquitoes only	
Chitnis	Method 1 infected	(*γ*_*h*_,*μ*_1*m*_,*ρ*_*h*_,*ψ*_*m*_,*σ*_*m*_)
	humans only	
	Method 1 infected	(*ψ*_*m*_,*γ*_*h*_,*μ*_1*m*_,*μ*_2*m*_,*ν*_*m*_,*σ*_*m*_)
	mosquitoes only	
	Method 2 infected	(*γ*_*h*_,*ψ*_*m*_,*σ*_*m*_,*ρ*_*h*_,*Λ*_*h*_,)
	humans only	
	Method 2 infected	(*σ*_*m*_,*μ*_1*m*_,*μ*_2*m*_,*ψ*_*m*_,*γ*_*h*_)
	mosquitoes only	

**Table 5:** Parameters of high sensitivity (greater than 5%) across all models

	**Ross**	**MacDonald**	**Anderson/May**	**McKenzie**	**Chitnis**
P(1,1)				*h*(5)*	*ψ*_*m*_ (8)*
P(1,2)				*h*(5)*, *DH*(5)*	*ψ*_*m*_ (8)*
P(1,3)				*h*(5)*, *DH*(16)*	*ψ*_*m*_ (8)*
P(2,1)		*μ*_2_(6)*, *a*(5)*	*μ*_2_(6)*, *a*(6)*	*h*(6)*, *IM*(5),	*μ*_1*m*_(20), *γ*_*h*_(12-14)
				*WN*(5-7), *DH*(5)*	*μ*_2*m*_(12-14), *σ*_*m*_(10)
				*d*(15)*, *f*_1_(15)*	*ψ*_*m*_(7)*, *Λ*_*h*_(6)*
					*ψ*_*h*_(6), *δ*_*h*_(5-6),
					*μ*_1*h*_(5), *ν*_*h*_(5), *ρ*_*h*_(5)*
P(2,2)	*a*(6)*,	*a* (7-8), *μ*_2_(8)*, *c*(5)*	*a* (8-9), *μ*_2_(5-9),	*DH*(11-44), *IM*(5),	
			*c* (6)*, *r*(5)*	*h*(6)*, *WN*(5-7),	
				*f*_1_(15)*, *d*(15)*	Same as above
P(2,3)	*a*(6-8), *c*(5)*,	*a*(10-12), *μ*_2_(7-10),	*a* (8-9), *μ*_2_(5-9),	*DH*(11-34), *IM*(5),	
	*μ*_2_(5)*	*c*(7)*, *r*(6)*, *b*(6)*	*c*(6)*, *r*(5)*, *b*(5-7)	*h*(6)*, *WN*(5-7),	
				*f*_1_(15)*, *d*(15)*	Same as above
P(3,1)			*μ*_2_(5)*	*h*(9)*, *WN*(5-9), *IM*(5)	*ψ*_*m*_(12)*
				*f*_1_(15)*, *d*(15)*	
P(3,2)			*μ*_2_(5)*	*h*(9)*, *WN*(5-9), *IM*(5)	*ψ*_*m*_(12)*
				*f*_1_(15)*, *d*(15)*, *DH*(88)*	
P(3,3)			*μ*_2_(5)*, *r*(5)*	*h*(9)*, *WN*(5-9), *IM*(5)	*ψ*_*m*_(12)*
				*f*_1_(15)*, *d*(15)*, *DH*(89)*	

Key: P(i,j) means parameter set i and initial conditions j. An asterisk indicates a parameter to which only one of $i_{h}^{*}$, $i_{m}^{*}$ was sensitive and change in the other was negligible. Numbers in parentheses indicate the largest (approximate) percent change in magnitude (in either $i_{h}^{*}$ or $i_{m}^{*}$) resulting from a 5% change in corresponding parameter value.

Final sensitivity rankings were derived from these results in two different ways from the nine rankings derived from calculations for each model. Method 1, $\left(\right)\sum \frac{1}{\text{rank}}))$, put more emphasis on specific, very high sensitivities and Method 2, $\left(\right)\sum \text{tot}-\text{rank}))$, placed more emphasis on consistent mid to high sensitivities where *tot* is the total number of parameters being tested. Table 1 shows the most sensitive parameters and their respective rankings for each model.

Rankings do not tell the whole story. In different models these rankings mean different things. For Chitnis, there are 17 parameters, and thus the top five listed in this table are very sensitive. Conversely, Ross has only five parameters total, so the lower ranked parameters shown in these tables are not necessarily very sensitive. Another way to understand which parameters are important is to compare the effect size. In Table 2 all parameters are given which, when varied by 5% gave variation in $i_{m}^{*}$ or $i_{h}^{*}$ of at least 5%, on a case by case basis across all nine choices of parameters and initial conditions. The approximate percent variation caused by altering the given parameter is given in parentheses. If no parameters cause a change of over 5% the entry is blank.

### Development of control trials

**Figure 4:**
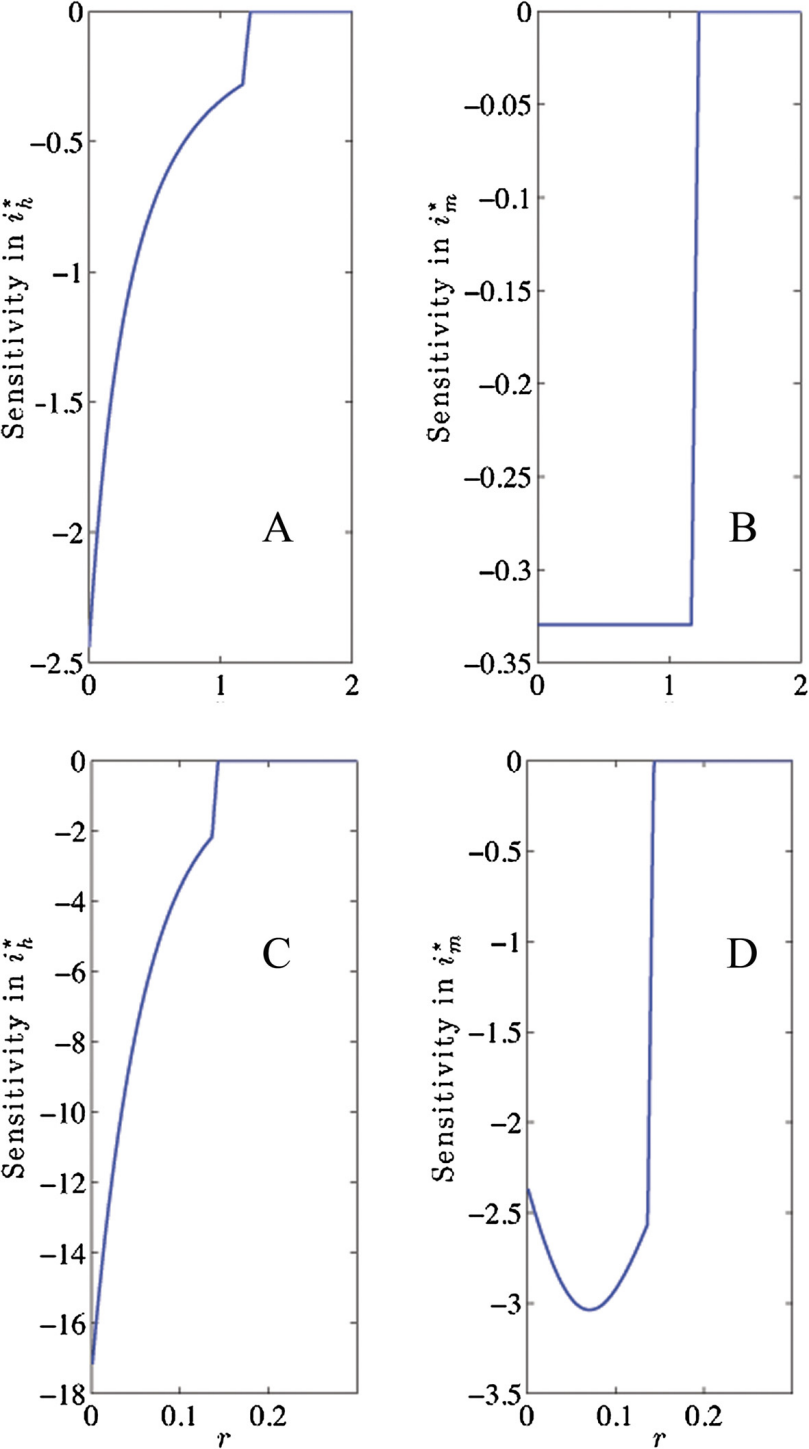
**Sensitivity profiles for recovery rate for two models.** Macdonald, Anderson and May sensitivity plots for recovery rate using parameter set 3 and “medium transmission” initial conditions, as in Table 5. **A.**$\partial i_{h}^{*}/\mathrm{\partial r}$*versusr*, Macdonald model, **B.**$\partial i_{m}^{*}/\mathrm{\partial r}$*versusr*, Macdonald model, **C.**$\partial i_{h}^{*}/\mathrm{\partial r}$*versusr*, Anderson/May model, **D.**$\partial i_{m}^{*}/\mathrm{\partial r}$*versusr*, Anderson/May model. Note the different profiles for the sensitivity of $i_{m}^{*}$ at low recovery rates.

Based on these rankings, three parameters were chosen for further study: bite rate, mosquito mortality rate, and human recovery rate. For these three, sensitivity profiles (*e.g.*$\partial i_{h}^{*}/\partial \alpha$ for parameter *α*) were computed across a range of parameter values for each of the choices of parameter sets and initial conditions. General trends were noted, along with seeming anomalies. Sensitivity profiles for these three parameters, for all five models and nine sets of baseline data as in Table 4, were studied with the goal of developing control trials that could further distinguish between models. Based upon the behaviour of the Macdonald and Anderson/May models shown in Figure 4, two models were chosen for control trials and comparison using the same parameter set.

**Figure 5:**
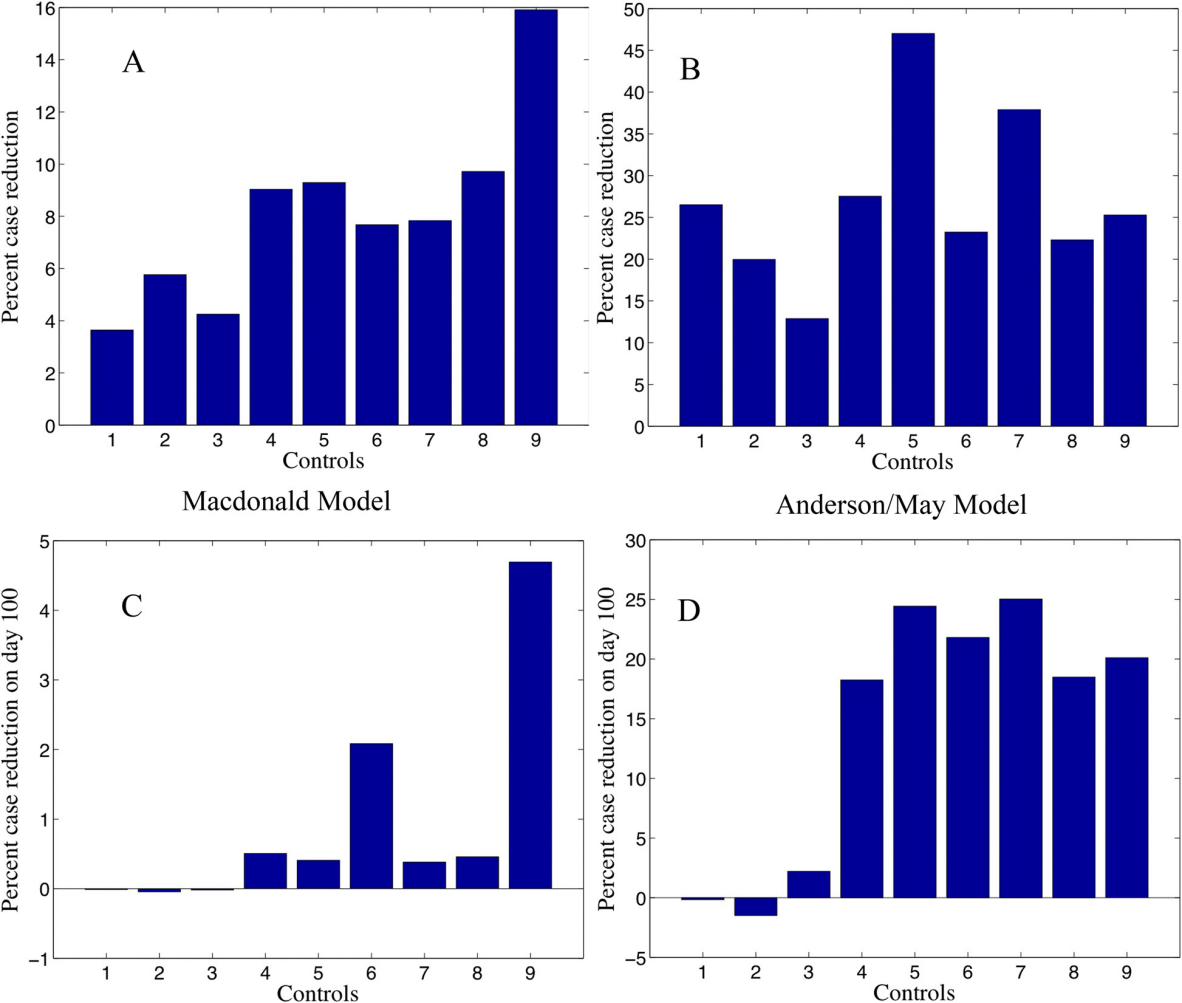
**Results of control trials for parameter set 3 and “medium transmission” initial conditions, as in Table **5**.A.** Percent case reduction overall resulting from nine control strategies, Macdonald model, **B.** Percent case reduction overall resulting from nine control strategies, Anderson/May model. **C.** Cases on day 100 resulting from nine control strategies, Macdonald model, **D.** Cases on day 100 resulting from nine control strategies, Anderson/May model. Left to right the numbered bars represent: 1) No intervention, 2) 50% reduction of bite rate for days 20–50, 3) doubling of mosquito mortality rate for days 20–50, 4) 50% reduction of bite rate for days 20–50 then doubling of human recovery rate for days 60–90, 5) doubling of human recovery rate for days 20–50 then 50% reduction of bite rate for days 60–90, 6) doubling of mosquito mortality rate for days 20–50 then doubling of human recovery rate for days 60–90, 7) doubling of human recovery rate for days 20–50 then doubling of mosquito mortality rate for days 60–90, 8) doubling of the mosquito mortality rate for days 20–50, followed by a 50% reduction of the bite rate, 9) 50% reduction of bite rate for days 20–50, followed by a doubling of the mosquito mortality rate for days 60–90.

A series of control trials of the Macdonald and Anderson/May models showed the predicted percent improvement in infected humans over 100 days. Typical interventions would include 1) spraying for mosquitoes, which would increase the mosquito mortality rate, 2) the use of bed nets, which would reduce the bite rate, and 3) screening for infected humans and treating them, which would increase the human recovery rate. As screening for infection and subsequent treatment is expensive compared to spraying or the use of bed nets, it is of practical use to know whether it’s effect could be enhanced by adjusting the timing of the intervention. Dynamical models are meant to shed light on time dependent phenomena, so the control trials chosen concentrate on the timing of these three types of intervention.The interventions simulated were: 1) No intervention, 2) 50% reduction of bite rate for days 20–50, 3) doubling of mosquito mortality rate for days 20–50, 4) 50% reduction of bite rate for days 20–50 then doubling of human recovery rate for days 60–90, 5) doubling of human recovery rate for days 20–50 then 50% reduction of bite rate for days 60–90, 6) doubling of mosquito mortality rate for days 20–50 then doubling of human recovery rate for days 60–90, 7) doubling of human recovery rate for days 20–50 then doubling of mosquito mortality rate for days 60–90, 8) doubling of the mosquito mortality rate for days 20–50, followed by a 50% reduction of the bite rate, 9) 50% reduction of bite rate for days 20–50, followed by a doubling of the mosquito mortality rate for days 60–90. The results of these numerical experiments are in Figure 5.

## Results

### Magnitude of human and vector disease prevalence at equilibrium

All models include the two infectious populations, *i*_*m*_ and *i*_*h*_ given as the proportion of mosquitoes infectious and the proportion of humans infectious, respectively. With comparable initial conditions and parameters, these five models give very different predictions of what happens at equilibrium, varying from 10 to 70 percent infected humans for parameter set 2 and initial condition 1. This variation is visible across a range of parameters, as illustrated in the examples in Figure 2. For all models the parameters expected to produce higher (or lower) rates of disease did so, with corresponding changes in the magnitude of the reproduction number.

### Consistency with respect to complexity of the model

For any of the nine sets of parameters and initial conditions, the equilibrium value for infected humans (or mosquitoes) always occurs in the same order for all models except McKenzie’s. The simplest model (Ross) has the most infected individuals. As the models come to contain more compartments and more complex expressions, a smaller percentage of infected humans is present at equilibrium. Thus, the second most complicated model (Macdonald) has fewer infected individuals at equilibrium, the third most complicated (Anderson/May) has even fewer and the most complicated model (Chitnis) has the lowest proportion infected ($i_{h}^{*}$) at equilibrium. Consistent with this observation, reproduction numbers decline as the model becomes more complex, with the exception of McKenzie, whose reproduction numbers did not behave consistently with respect to the other models.McKenzie’s model has the same number of compartments as the Anderson/May model, but its equilibrium values do not respect any particular ordering according to its complexity among the five models considered. Furthermore, the McKenzie model has the unusual feature that in some cases (parameter set 2 and initial conditions 1) the infected mosquito equilibrium is high, second only to Ross, while the infected human equilibrium is low: just above that of Chitnis. This behaviour is visible as outliers to the general trend in Figure 3A.

### Consistency with respect to intuition and data

Intuition suggests that the proportion of infected humans should rise with the bite rate and decline with increased mosquito mortality. Figure 2 shows that all of the models behave this way at equilibrium. Intuition also suggests that, at equilibrium, as the proportion of infected mosquitoes rises, so would the proportion of infected humans. Figure 3A shows this to be the case for four out of the five models.

Figure 3B shows $i_{h}^{*}$*versus* the entomological inoculation rate (EIR, the product of the man-biting rate and $i_{v}^{*}$). The range of observed EIR found in the literature is from one to one thousand infective bites per year (or 2.7 infective bites per day) [10]. Note that only one of the models exceeds this rate, for two choices of parameters. Beier *et al.*[10] conducted a review of many studies, and found a log-linear dependence of malaria prevalence on the entomological inoculation rate. The relationship they found is plotted in Figure 3B against output from all five models analysed in this paper. Two of the models fit the observed relationship quite well and two fit poorly, with one mixed result that fit well for six of the nine simulations.

It is intuitive that the disease prevalence in humans should be related to the overall man-biting rate. For all models the man-biting rate was calculated at equilibrium. For the Ross, Macdonald, Anderson/May and McKenzie models, this is given by the bite rate multiplied by the ratio of mosquitoes to humans at equilibrium. For Chitnis it is represented by the modified Hill function at equilibrium (see the equation in Table 2). Figure 3C shows the relationship between the man-biting rate and $i_{h}^{*}$ across all models and parameter sets. One model scaled significantly differently from the others, and is removed from the re-scaled plot in Figure 3D. This figure shows that one of the models did not agree with intuition.

The quantities displayed in Figure 3, (percent of mosquitoes infected, entomological inoculation rate, and man-biting rate), are all measurable to some extent in the field. The summary data in Beier *et al.*[10] represents the sort of data to which models should be compared. The correlation (or lack thereof) between these potentially measurable quantities and disease prevalence distinguishes the various models and represents not only a point of comparison between models, but also a point of comparison against data sets.

### Parameters to which models were most sensitive

The parameters to which all five models are most sensitive are, with few exceptions, common to all five models, as is seen in Table 1. They are almost all mosquito parameters which are to some extent subject to human control (bite rate, birth rate, death rate) and also recovery rate of humans, which is under human control. Although all of these models are nonlinear, there does not appear to be any disproportionately large response to parameter changes. Small changes in even the most important parameters resulted in at most moderate changes in equilibrium values, across all five models and all nine choices of initial conditions and parameters.

Response to change in parameters seems to rise with the complexity of the model. In Chitnis, many of the important parameters have to do with construction of the term describing bite rate (which is given as a modified Hill function). Each of these parameters separately has a big effect. Together these effects could either cancel out or magnify each other. Because the sensitivity rankings for the Chitnis model vary markedly with parameter set, any existing patterns in these rankings are obscured in the rankings across all parameter sets presented in Table 1. Table 2 shows all parameters that gave a response greater than or equal to 5% when varied by 5%. With the exception of the McKenzie model, models were sensitive to more parameters when measured from the second set of parameters as a baseline (reflecting a low transmission rate).

The only models that include mosquito birth rate explicitly are Chitnis and McKenzie. In the Chitnis model, this parameter (*ψ*_*m*_) has a large effect for all sets of parameters and initial conditions (7–12% response for a 5% change in birth rate, even in high disease prevalence conditions). Table 2 shows that the Chitnis model is sensitive to many parameters when measured from the baseline of the second parameter set, but sensitive to *ψ*_*m*_ in all trials.

### Sensitivity profiles as a parameter varies across its range

All models show reduced sensitivity to bite rate at high bite rates. With the exception of McKenzie, the models show increased sensitivity to bite rate for *R*_0_ near and greater than the critical threshold, as expected. In all models the sensitivities of $i_{m}^{*}$ and $i_{h}^{*}$ to bite rate are positively correlated. This may seem intuitive but it is not the case for some of the other parameters.

Models disagree on the sensitivity profile for mosquito mortality. The sensitivities of $i_{m}^{*}$ and $i_{h}^{*}$ are not necessarily correlated as the death rate changes. The disagreement is across models and within models as parameters vary, with no parameter set on which they all agree. For comparable parameter sets one model may show the sensitivities of both $i_{h}^{*}$ and $i_{m}^{*}$ dropping as the death rate approaches the critical value, while another may show heightened sensitivity of $i_{h}^{*}$ as the death rate approaches critical. Figure 4 shows an example of this behaviour in the Anderson/May model. In the Ross model, the sensitivity of $i_{m}^{*}$ drops steadily while the sensitivity of $i_{h}^{*}$ remains constant until the critical value. In the Macdonald and Anderson/May models the sensitivity of $i_{m}^{*}$ drops steadily while the sensitivity of $i_{h}^{*}$ rises until the critical value is reached, then drops. In the McKenzie model, the sensitivity of $i_{m}^{*}$ remains constant while the sensitivity of $i_{h}^{*}$ rises for parameter set 1, but for the other two parameter sets the model behaves like the Macdonald and Anderson/May models.

Note that Chitnis uses both a density dependent death rate and a density independent death rate. For the analysis here the density independent rate was chosen as it corresponds both to normal mosquito control measures and to the rate constants used in the other models.In the Chitnis model the sensitivities of $i_{m}^{*}$ and $i_{h}^{*}$ were sometimes positively and sometimes negatively correlated as mosquito mortality varied, with no discernible pattern across the nine runs.

Of all control measures, treatment that increases human recovery rate is probably the most expensive to implement. It is particularly important to know when a modest treatment effort is likely to have a large impact on the overall disease burden. Unfortunately, the models studied here provide no consistent guidance on this matter. This is another situation where the sensitivities of $i_{h}^{*}$ and $i_{m}^{*}$ are sometimes negatively correlated as the recovery rate increases and approaches the critical value where *R*_0_ drops below one. The Anderson/May model in particular displays this behaviour for parameter set 3 and initial conditions 2, as shown in Figure 4, with the Macdonald model shown for comparison. These two models differ only in the presence of an “exposed” category for humans and a time delay for that exposure (see Table 1). On the basis of this unusual behaviour, this pair of models and these parameters and initial conditions were chosen for control experiments, described below.

### Control experiments

The behaviour of the sensitivity profile is important for control purposes. Some of these models suggest that mosquito mortality has a large, noticeable effect on disease prevalence for *R*_0_ near, but greater than 1. Others suggest only modest change in disease prevalence until the critical value is achieved. Knowing which type of behaviour to expect from mosquito mortality is a useful way to distinguish various models, although it is difficult to quantify directly. For example, the Macdonald and Anderson/May models are quite similar in approach. Yet because of the different sensitivity profiles (such as those shown in Figure 4) the models would be likely to suggest quite different control strategies based on alternative timing of treatment.

These two models were chosen for comparison in the control trials. It is important to note that these control trials measure the performance of the models before they arrive at equilibrium. They are thus measuring a feature of the model not captured by other measures described in this paper.

The results are shown in Figure 5. Timing of an intervention may be a key factor in its success. One interesting contrast in Figure 5 is between the results of first doubling the mosquito death rate and then doubling the human recovery rate *versus* the reverse order of control methods (bars 8 & 9). Notice that the Anderson/May model predicts a much larger reduction in infected humans if mosquito mortality is doubled first, followed by doubling human recovery rates, whereas the Macdonald model shows little difference between these two sequences. So, although it is difficult to quantify directly the difference between the sensitivity profiles of these two models, it is easy to quantify the effects of such a difference in control trials.

## Conclusions

### General observations

For all models, the parameter sets representing high/low disease transmission did in fact lead to higher/lower percent of infected humans ($i_{h}^{*}$) at equilibrium. In general, as the complexity of the model (the number of quantities tracked) increased, the percent of infected humans at equilibrium decreased. For most models, the percent of infected humans at equilibrium was correlated with the percent of infected mosquitoes and also the man-biting rate. The McKenzie model tended to behave quite differently from the other four models under the benchmark tests. However, the mere fact that the McKenzie model was an outlier in these analyses is insufficient to draw conclusions about which model is the “best” model for malaria. Validating these models against data is critical for determining which model best describes malaria dynamics.

Parameters introduced in more complex models tend to be less important than the ones included in all models, from the point of view of sensitivity. The early, simple models capture most of the control dynamics that are likely to work. This is good because they tend to include parameters one might be able to measure.

### Benchmark tests for any model of malaria transmission

Profoundly different behaviours and sensitivity profiles of these five models suggest the need for benchmark tests that will allow models to be compared easily against each other and, more importantly, against data sets. Figure 3 suggests two of these. Scatterplots, across a variety of parameter choices, of the proportion of infected humans at equilibrium *versus* both the proportion of infected mosquitoes and man-biting rate give a quick way to compare models. Even better, models may be compared against data sets using this method, and tuned accordingly. For example, incidence of malaria is correlated with vector density and resulting entomological inoculation rate as described by Beier *et al.*[10], Elissa *et al.*[11] and Smith *et al.*[12].

Different control strategies are suggested by different patterns of sensitivity. For example, some models suggest that although the sensitivity of $i_{m}^{*}$ drops with increased mosquito death rate, the sensitivity of $i_{h}^{*}$ increases. If this is correct, a small increase in adulticide could yield big gains even when mosquito populations are already low. Therefore a few simulations of control measures also serve to distinguish models.Note that these benchmarks suffice to distinguish among all but the two simplest models (Ross and Macdonald), which differ only in magnitude of the predicted disease prevalence. Furthermore, if data can be shown to agree with intuition, as in the case of the entomological inoculation rate, then there will be a strong basis for expecting models to agree with the trends displayed in Figures 2 and 3.

The list below summarizes these suggestions.

#### (1) Consistency of model performance

Evidence for this can be inferred by comparing the magnitude of infected human and mosquito populations at equilibrium with the four models in this paper that showed consistency with respect to each other (Ross, Macdonald, Anderson/May and Chitnis), across a range of parameters and initial conditions, as in Figure 2. Generally one would expect a model with more compartments to predict a smaller percentage of population in each compartment. This benchmark would help the modeler identify unusual behaviour, such as simultaneous low disease prevalence in humans and high disease prevalence in vectors, as seen in the McKenzie model.

#### (2) Comparison with data

Predicted entomological inoculation rate at equilibrium for a variety of reasonable parameter sets can be compared with the range described by Beier *et al.*[10]. Failure to predict EIR values in this range would indicate that a model was calibrated poorly.

The relationship of infected humans to entomological inoculation rate at equilibrium may be compared with the data based model from Beier *et al.*[10] as parameters vary, as in Figure 3B. This data set, gleaned from a meta-analysis, gives modelers a unique opportunity to test their model against the real world. The simpler Ross and Macdonald models fit the data fairly well. More complex models should preserve this general relationship. Although equilibrium values across a variety of parameter sets were used to generate the plots in Figure 3, a single model with a well justified parameter set could be tested in the same way as time varies.

#### (3) Comparison with intuition

Relationship of infected humans to man-biting rate at equilibrium, across a range of parameters as in Figures 3C and 3D. As the figure shows, some models do not produce high rates of infection even with the man-biting rate grows large. Similarly, the relationship of infected humans could be compared to infected mosquitoes at equilibrium, across a range of parameters, as in Figure 3A. A general positive correlation would be expected. Data sets similar the the one for EIR in Beier *et al.*[10] would be far better.

#### (4) Comparison of most influential parameters

Ranking the sensitivity of infected mosquitoes and humans at equilibrium to all parameters using methods 1 and 2, as in Table 1, and identification of parameters to which infected mosquitoes and humans at equilibrium are particularly sensitive, as in Table 2, give an indication of what features control most of the predictive value of a model. The top parameters were fairly consistent across models. The introduction of a new parameter that outranks bite rate, mosquito mortality or birth rate, would simultaneously be quite interesting and also a cause for concern, and may require further justification or explanation. Also, a parameter that appears to exhibit undue importance may simply be calibrated poorly and may be the cause of discrepancies in tests such as those in Figure 3. The behaviour of sensitivity of infected mosquitoes and humans to important parameters near critical values of *R*_0_, for a series of baseline parameter tests, as in the example in Figure 4, gives additional information. Variations in this profile allow modelers to search for parameter sets that are likely to respond quite differently to control measures.

#### (5) Response to malaria control measures

Comparison of control measures that vary the sequence of interventions, especially for parameter sets with unusual behaviour, as illustrated in Figure 5. These tests measure properties of a system not yet at equilibrium and, as such, are representative of most natural systems. Models in which timing and order of interventions make a big difference are worth further investigation, and understanding scenarios in which timing and order of interventions matter would be of practical use.

### A note on mosquito dynamics and spatial models

In all of the models considered, mosquito dynamics are either represented trivially (as a constant recruitment rate) or arrive at equilibrium quickly. On the other hand, many parameters that are set to a constant value in these models are actually dependent on mosquito population. These are indicated in Table 4 by an asterisk next to them. The various authors have implicitly built mosquito dynamics into parts of their models without representing the lifecycle directly. Each author has done so differently. Indeed, it is almost surprising that the sensitivity profiles for bite rate are so consistent given these very different choices of representation of mosquito population, and less surprising that the other sensitivity profiles are quite different from each other. The various ways in which mosquito dynamics are implicitly built into these models is likely to be a large part of the reason for the different behaviours of these models.

Lindblade *et al.*[13] found a correlation between vector abundance, as measured by indoor resting density, and malaria incidence in a highland region of Uganda. Models that incorporate mosquito population dynamics could be tested to see to what degree and under what conditions the model predicts such a correlation. These authors note that increased rainfall precedes an increase in vector density with a characteristic time lag. Models incorporating mosquito dynamics with varying larval habitat should also display similar properties.

However, introducing mosquito dynamics increases the complexity of models. It may be possible that, once good insect models are coupled to malaria transmission models, some of the inconsistencies between models, as noted in this paper, may diminish. Before coupling mosquito dynamics to a malaria model, mosquito population models must also be tested against each other. In short, a set of benchmarks for mosquito dynamics should be developed to compliment the benchmarks for malaria dynamics suggested in this paper.

This recognition of the importance of mosquito dynamics suggests that models need to include the effects of spatially distributed and temporally evolving environmental conditions (as many are now doing, [14-23]). Before extending non-spatial models to a spatial domain, it is important to understand how the model behaves in a series of tests such as those suggested here, rather than just understanding how it is constructed, otherwise it is possible that disagreements between spatial models may just reflect different dynamics of the underlying, non-spatial model.

### To what extent should one trust models?

All of the models discussed in this paper, as well as many more in the literature, are conceptually reasonable. Yet they produce very different results when carefully calibrated to correspond to each other and run through a series of tests. It is perfectly natural to wonder if one should throw all the models out.

It is important to remember that there are many points on which all of the models tested here agree. Based on the sensitivity analysis, they all agree on the importance of bite rate, recovery rate, and mosquito mortality rate. They all show similar trends in disease prevalence as these rates change. If one did not hope for absolute numbers, one could take comfort in the similarity of these gross trends. Good qualitative behaviour is ultimately unsatisfying though, as these trends are already widely accepted and do not require our models to justify them.

To take the benchmarks described here seriously, one would have to admit that the simpler models (Ross, Macdonald, Anderson/May) seem to be doing a better job of matching data and heuristics than the more complicated models (Chitnis, McKenzie). These results, therefore, suggest a strategy of adding new features one at a time to models, and retesting them against the benchmarks to see that overall agreement with data and heuristics has not been lost. Both small problems of calibration and larger conceptual difficulties could be identified in this way. If models are to be useful, field researchers will need to assemble data sets against which they may be compared and tested, such as the one provided by Beier *et al.*[10]. In particular, modelers need data sets either justifying or correcting all of the heuristics described in Figures 2 and 3.

However, the results of the control trials on the Macdonald and Anderson/May models should also be taken seriously. These two models performed consistently although not identically on benchmarks 1-4, yet gave very different answers on benchmark 5. When to undertake control measures is a basic question that dynamical models ought to be able to answer (correctly). Appropriate timing of an intervention can increase its impact, which is particularly important if the intervention is expensive. It is clear at this point that it would be unwise to trust a model to answer this question. The results of this paper suggest two strategies for bringing various models into alignment with each other on this point. One is to include basic, validated mosquito dynamics, so that models are not forced to infer these in inevitably different ways. The second is to calibrate models against better data sets if possible, in advance of attempting to make predictions.

Models are usually compared based on what quantities are modeled, how their relationships are expressed, and the qualitative nature of output in terms of fixed points and bifurcations. This paper adds additional useful points of comparison based on the performance of a model in relation to itself, to data, and to a few other models.

The benchmarks suggested in this paper are meant to serve modelers in the future. Benchmark 1 is a test of internal consistency. Benchmark 2 is a comparison with data. Benchmark 3 is a comparison with intuition which could perhaps be expressed in a data set. Benchmark 4 gives clues as to how to adjust a model appropriately or troubleshoot unusual behaviour. Benchmark 5 asks whether a model is capable of distinguishing among a series of control strategies, and under what conditions. Together these represent five tools modelers can use to improve the performance of their models in various ways.

## Competing interests

The authors declare that they have no competing interests.

## Authors’ contributions

DW designed the study, drafted the manuscript, collaborated on analysis of numerical experiments. BS conducted numerical experiments, collaborated on analysis of numerical experiments. All authors contributed to the writing and revision of the manuscript. XS, JC, and AG provided geographic, entomological and epidemiological perspective. All authors read and approved the final manuscript.

## References

[B1] Baumrin E Drexinger J Sotsky J Wallace D **Control of West Nile Virus by insecticide in the presence of an avian reservoir** Biomat 2010: International Symposium on Mathematical and Computational Biology, Rio de Janeiro, Brazil, 24–29 July 2010 2011 Singapore: World Scientific Publishing Company 126 145

[B2] Mandal S Sarkar RR Sinha S **Mathematical models of malaria-a review** Malar J 2011 10 202 21777413 10.1186/1475-2875-10-202 PMC3162588

[B3] Ross R The prevention of malaria, 2nd edition 1911 London, UK: Murray

[B4] Smith DL Battle KE Hay SI Barker CM Scott TW McKenzie FE **Ross, Macdonald, and a theory for the dynamics and control of mosquito-transmitted pathogens** PLoS Pathog 2012 8 e1002588 22496640 10.1371/journal.ppat.1002588 PMC3320609

[B5] Perkins TA Barker CM Niu T Chaves LF Ellis AM George DB Le Menach A Pulliam JR Bisanzio D Buckee C Chiyaka C Cummings DA Garcia AJ Gatton ML Gething PW Hartley DM Johnston G Klein EY Michael E Lindsay SW Lloyd AL Pigott DM Reisen WK Ruktanonchai N Singh BK Tatem AJ Kitron U Hay SI Scott TW Reiner RC Jr **A systematic review of mathematical models of mosquito-borne pathogen transmission 1970–2010** J R Soc Interface 2013 10 20120921 23407571 10.1098/rsif.2012.0921 PMC3627099

[B6] Chitnis N Cushing J Hyman J **Bifurcation analysis of a mathematical model for malaria transmission** SIAM J App Math 2006 67 24 45

[B7] Macdonald G The Epidemiology and Control of Malaria 1957 Oxford, UK: Oxford University Press

[B8] McKenzie FE Samba EM **The role of mathematical modeling in evidence-based malaria control** The Am J Trop Med Hyg 2004 71 2 Suppl 94 PMC2518450 15331824

[B9] Anderson RM May RM Anderson B Infectious Diseases of Humans: Dynamics and Control 1991 Oxford & New York: Oxford University Press

[B10] Beier JC Killeen GF Githure JI **Short report: entomologic inoculation rates and Plasmodium falciparum malaria prevalence in Africa** Am J Trop Med Hyg 1999 61 109 113 10432066 10.4269/ajtmh.1999.61.109

[B11] Elissa N Migot-Nabias F Luty A Renaut A Toure F Vaillant M Lawoko M Yangari P Mayombo J Lekoulou F Tshipamba P Moukagni R Millet P Deloron P **Relationship between entomological inoculation rate, **** *Plasmodium falciparum * **** prevalence rate, and incidence of malaria attack in rural Gabon** Acta Trop 2003 85 355 361 12659973 10.1016/s0001-706x(02)00266-8

[B12] Smith D Dushoff J Snow R Hay S **The entomological inoculation rate and Plasmodium falciparum infection in African children** Nature 2005 438 492 495 16306991 10.1038/nature04024 PMC3128496

[B13] Lindblade KA Walker ED Onapa AW Katungu J Wilson ML **Highland malaria in Uganda: prospective analysis of an epidemic associated with El Niño** Trans R Soc Trop Med Hyg 1999 93 480 487 10696401 10.1016/s0035-9203(99)90344-9

[B14] Hoshen MB Morse AP **A weather-driven model of malaria transmission** Malar J 2004 3 32 15350206 10.1186/1475-2875-3-32 PMC520827

[B15] Ermert V Fink AH Jones AE Morse AP **Development of a new version of the Liverpool Malaria Model. I. Refining the parameter settings and mathematical formulation of basic processes based on a literature review** Malar J 2011 10 35 21314922 10.1186/1475-2875-10-35 PMC3055220

[B16] Jones AE Morse AP **Application and validation of a seasonal ensemble prediction system using a dynamic malaria model** J Climate 2010 23 4202 4215

[B17] Jones A Morse A **Skill of ENSEMBLES seasonal re-forecasts for malaria prediction in West Africa** Geophys Res Lett 2012 39 23 [http://onlinelibrary.wiley.com/doi/10.1029/2012GL054040/full]

[B18] Lunde TM Korecha D Loha E Sorteberg A Lindtjørn B **A dynamic model of some malaria-transmitting anopheline mosquitoes of the Afrotropical region. I. Model description and sensitivity analysis** Malar J 2013 12 28 23342980 10.1186/1475-2875-12-28 PMC3664083

[B19] Lunde TM Balkew M Korecha D Gebre-Michael T Massebo F Sorteberg A Lindtjørn B **A dynamic model of some malaria-transmitting anopheline mosquitoes of the Afrotropical region. II. Validation of species distribution and seasonal variations** Malar J 2013 12 78 23442727 10.1186/1475-2875-12-78 PMC3653715

[B20] Bomblies A Duchemin JB Eltahir EA **Hydrology of malaria: model development and application to a Sahelian village** Water Resour Res 2008 44 12 [http://onlinelibrary.wiley.com/doi/10.1029/2008WR006917/full]

[B21] Bomblies A Duchemin JB Eltahir EA **A mechanistic approach for accurate simulation of village scale malaria transmission** Malar J 2009 8 1 12 19799793 10.1186/1475-2875-8-223 PMC2761400

[B22] Tompkins AM Ermert V **A regional-scale, high resolution dynamical malaria model that accounts for population density, climate and surface hydrology** Malar J 2013 12 65 23419192 10.1186/1475-2875-12-65 PMC3656787

[B23] Eckhoff PA **A malaria transmission-directed model of mosquito life cycle and ecology** Malar J 2011, **10**. [http://www.malariajournal.com/content/10/1/303] 10.1186/1475-2875-10-303 PMC3224385 21999664

